# Numerical Analyses of Entropy Production and Thermodynamics Exergy on a Hydrogen-Fueled Micro Combustor Featuring a Diamond-Shaped Bifurcated Inner-Tube Structure for Thermophotovoltaic Applications

**DOI:** 10.3390/e27020114

**Published:** 2025-01-24

**Authors:** Faisal Almutairi

**Affiliations:** Department of Mechanical Engineering, College of Engineering, King Faisal University, Al-Ahsa 31982, Saudi Arabia; falmutairi@kfu.edu.sa

**Keywords:** entropy, exergy, heat transfer, renewable energy, hydrogen combustion, micro combustor, thermodynamics

## Abstract

To improve the heat transfer mechanisms from the thermal energy to the walls, the current work presents a new structure for a micro combustor fueled by hydrogen featuring a diamond-shaped bifurcated inner-tube configuration. For this purpose, a series of three-dimensional (3D) numerical analyses are conducted to investigate the effects of the length of the diamond-shaped structure, width of inner flame channels, inlet equivalence ratio, and hydrogen volume flow rate on the key performance and thermodynamic parameters. In comparison to the conventional design, the outcomes reveal that the proposed configuration exhibits remarkable improvements in energy conversion efficiency, as it reduces the mean exhaust gas temperature by 585.98 K and boosts the exergy and radiation efficiencies by 7.78% and 14.08%, respectively. The parametric study of the design parameters indicates that elongating the diamond-shaped structure and widening the inner flame channels enhance the thermal dynamics and consequently improve the rates of heat absorption by the walls. The increase in the hydrogen volume flow rates feeds the system with additional energy and, therefore, advances the average wall temperature and its uniformity across the external surface. Nevertheless, it also reduces system efficiency due to the limited capacity of the micro combustor to utilize a large energy input along with the high magnitude of entropy production resulting particularly from the mechanism of chemical entropy generation. Operating under a stoichiometric condition balances hydrogen and oxygen in the premixed charge, achieving optimal thermal performance for the micro combustor.

## 1. Introduction

The utilization of fossil fuels in combustion-based applications releases a significant quantity of greenhouse gases [1,2], contributing to critical environmental challenges, including global warming [3,4]. Combined with the rapid depletion of fossil fuel reserves [5], these issues have accelerated global efforts to transition toward cleaner energy sources. In recent decades, hydrogen has garnered considerable interest among researchers and is increasingly viewed as a viable pathway for achieving sustainable energy transitions [6]. As a zero-carbon fuel, hydrogen combustion notably emits less environmental pollution than conventional fuels [7]. In addition, hydrogen exhibits high performance owing to its high flame propagation and diffusivity rate, which enhance combustion efficiency [8]. The wide flammability range of hydrogen also improves fuel economy by enabling the use of lean or even extra-lean mixtures. However, a key drawback of hydrogen combustion is its emission of nitrogen oxides (NO_x_), which is exacerbated by the high-temperature environment characteristic of hydrogen combustion [9]. Unlike large-scale systems, such as industrial boilers and diesel engines, micro-scale applications like micro thermophotovoltaic (MTPV) systems operate with extremely low mass flow rates, resulting in considerably lower NO_x_ emissions [10,11].

MTPV technology is a power generation system that has recently gained attention due to its lack of moving parts and its straightforward manufacturing and assembly processes [12]. Furthermore, combustion-based micro power generators offer several advantages over batteries, such as higher energy density and greater mass and heat transfer coefficients [13,14,15]. In MTPV systems, thermal energy from combustion heats the walls of the micro combustor, which then radiates high-energy photons toward the photovoltaic (PV) cells to generate electricity [16,17]. Therefore, the micro combustor serves as the primary energy source, implying that it is the core component in MTPV systems. Hence, improving the key thermal parameters of the micro combustor ensures an enhancement of radiation efficiency, and consequently, more electricity is generated. Various factors are important to consider in investigating such a system; however, the critical metrics that indicate further advancements in the MTPV system’s power density output and emitter efficiency are the outer wall temperature and the even variation of temperature across the walls, which largely depends on the heat absorption rate of the wall. Several parameters control the heat transfer mechanisms, such as the thermal conductivity of material and fluid, roughness and area of the contacted surface, fluid velocity, fluid viscosity, the emissivity of the surface, and fluid disturbance intensification.

Numerous efforts have been made to advance the heat transfer mechanisms of micro combustors through various techniques, such as incorporating cavities [18,19], inserting porous media (PM) [20,21,22], and using alternative fuels like propane [23,24], methane [25,26], and ammonia [11,27]. Many investigations have also focused on improving heat transfer capacity by redesigning the geometric structure of micro combustors. These studies have demonstrated that the optimization of the micro combustor structure can considerably advance radiation efficiency. For example, Cai et al. [28] examined the effects of employing staggered bluff bodies on the heat-related parameters of a hydrogen-fueled meso-combustor. The authors highlighted that inserting staggered bluff-bodies generates longitudinal vortices, which improve the mixing process and energy conversion efficiency and, therefore, advance the mean wall temperature by 73 K. Zhao et al. [29] examined the heat transfer parameters of a hydrogen-fueled meso-combustor with a design embedded double-channel Y-shaped internal fins. It was reported that increased inlet velocity promotes higher mean wall temperature, radiation rate, and exergy. In addition, the unity equivalence ratio optimizes thermal efficiency. Lachraf and Ameur [30] investigated the effects of a new micro combustor structure with four equally spaced trapezoidal ribs on the inner wall. It was found that the new design enhances recirculation zones and consequently increases the residence time of flow. For this, an increase in the mean wall temperature was achieved by roughly 46 K. In the same vein, Rong and Zhao [31] conducted thermodynamic and entropy generation analyses of a meso-combustor featuring Tesla-valve-type flow channels. Their findings demonstrated that the optimal case with the Tesla valve effectively increases wall temperature by 72.6%. In addition to these studies, many new designs of micro combustors have been proposed in the literature, as can be found in [32,33].

Through the proposed configuration highlighted above, the bifurcated channel-like structure has been employed in different studies in the literature and demonstrated to intensify the fluid disturbance. This results from the induction of a greater complex flow pattern by the bifurcated configuration, which leads to further improvements in the heat exchange between the fluid and solid walls [34]. Huang et al. [35] investigated the effects of a heat exchanger featuring bifurcated bare tubes in a water-based hybrid variable refrigerant flow system on the system efficiency. The results revealed that the proposed bifurcated design increases the heat transfer capacity of the air side by 117% and, in addition, decreases the air-side pressure drop by 61%, the total pumping power by 65%, the total material volume by 75%, and the envelope volume by 65%. Yu at el. [36] performed a numerical analysis to compare the effects of a constructal bifurcation filler and porous media on the flow and heat transfer process. It was reported that the constructal bifurcation filler intensifies the fluid mixing and results in a better temperature uniformity of fluid due to the generation of longitudinal whirling flow with multiple vortexes. Moreover, the new proposed structure was found to reduce the flow resistance and to advance the energy conversion efficiency. Safari et al. [37] experimentally and numerically examined how the paraffin inside the shell-and-tube heat exchanger, employing straight and bifurcated fin designs, affects the melting process. The outcomes indicated that the tree-like structure enhances the thermal performance of the system at a higher degree compared to the straight fin configuration, consequently yielding a decrease in the maximum melting temperature of more than 50%. Similarly, Luo et al. [38] performed an investigation into the melting process of phase change materials in a heat exchange featuring fractal fins with two bifurcation levels. The results revealed that inserting two levels of tree-like fractal fins decreases the melting time by 68%, in contrast to the traditional fractal fin. Sciacovelli et al. [39] studied the heat-related parameters of a double-pipe heat exchanger incorporating Y-shaped fins with one and two tree-like structures. They reported that the Y-shaped fins with one and two bifurcations yield an improvement of the thermal performance by roughly 6% and 24%, respectively, compared to the conventional configuration.

Despite extensive efforts recounted in the literature to improve the thermal performance of micro combustors through new design configurations, there is a lack of investigation of the effects of employing the bifurcated configuration on the thermal performance of micro combustor applications. As there remains substantial potential for further improvement and exploration of how the bifurcated structure can affect the thermal dynamics of flow in micro combustors, this paper introduces a new design configuration of a hydrogen-fueled micro combustor featuring a diamond-shaped bifurcated inner-tube structure. Through numerical analysis, the research reported here examines the effects of the new configuration on the key thermodynamics and performance parameters. Section 2 outlines the design of the new micro combustor and details the computational framework, including the combustion modeling approach, initial and boundary conditions, and other numerical sub-models. In addition, mesh sensitivity analysis and validation processes are presented in this section. Section 3 describes the impacts of the diamond-shaped design as opposed to the traditional one, the length of the diamond-shaped structure, the widths of the inner tubes, the volume flow rate, and the inlet equivalence ratio. Section 4 provides an overview of the key observations concluded from the research.

## 2. Numerical Methodology

### 2.1. Geometric Model

**Figure 1:**
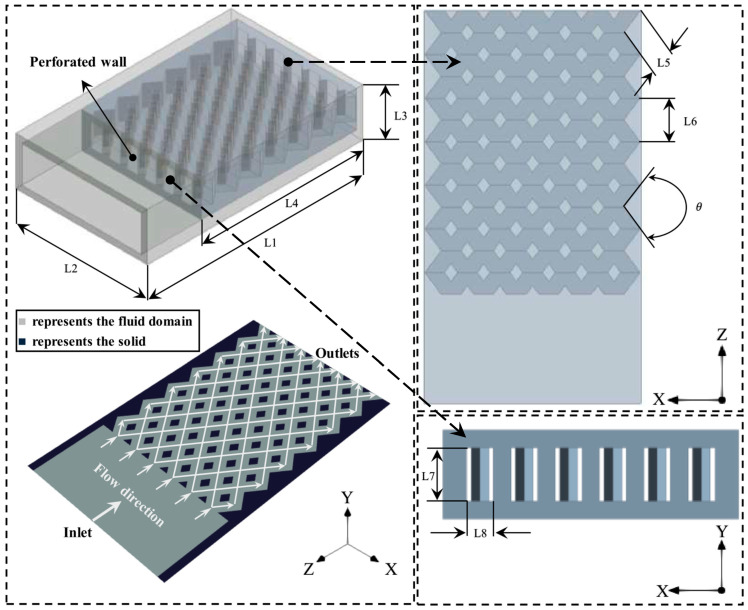
Schematic representation of the diamond-shaped bifurcated inner-tube micro combustor configuration. L stands for length, θ stands for angle.

The diamond-shaped bifurcated inner-tube micro combustor is depicted in Figure 1 and constructed using ANSYS Design Modeler. Table 1 lists the dimensions of all the test cases (note that the thickness of external walls is 0.5 mm). The potential of the new configuration lies in two key design points. The first is the middle-perforated wall, which acts as the holder of the high-temperature flame near the micro combustor inlet. This greatly elongates the dwell time of the high-thermal energy and consequently improves the heat absorption rate by the outer wall. The second is the diamond-shaped bifurcated inner tubes, in which each flame path branches into two paths and then merges again in a repeatedly equidistant manner. This structure has an effective means of evenly distributing heat to the external walls to enhance the uniformity of the mean wall temperature. The combustor is composed of steel material.

### 2.2. Governing Equations

The numerical investigations were conducted using the computational fluid dynamic (CFD) software ANSYS Fluent R1 2023 [40]. The governing equations solved during the simulation execution time are as follows:

Mass:

(1) $$\nabla \cdot \left(\rho \vec{v}\right)=0$$

Momentum:

(2) $$\rho \vec{v}\cdot \nabla \vec{v}=-\nabla P+\nabla \cdot \left(\vec{\tau }-{\vec{\tau }}^{\prime }\right)$$

where

(3) $$\vec{\tau }=\mu \left[\nabla \vec{v}+{\left(\nabla \vec{v}\right)}^{T}-\frac{2}{3}\nabla \vec{v}Ι\right]$$

Energy:

(4) $$\nabla \cdot \left(\vec{v}\left(\rho E+p\right)\right)=\nabla \cdot \left(k_{eff}\nabla T-\sum_{j}h_{j}{\vec{J}}_{j}+\left(\vec{\tau }\cdot \vec{v}\right)\right)+S_{h}$$

Species:

(5) $$\nabla \cdot \left(\rho \vec{v}Y_{i}+{\vec{J}}_{i}\right)=R_{i}$$

where ρ stands for the density, P stands for the static pressure, $\vec{v}$ stands for the velocity vector, ${\vec{\tau }}^{\prime }$ stands for the Reynolds stress, $\vec{\tau }$ stands for the viscous stress, Ι stands for the unit tensor, μ stands for the molecular viscosity, $k_{eff}$ stands for the effective conductivity, E stands for the total energy of the fluid, $h_{j}$ stands for the enthalpy of species j, T stands for the temperature, $S_{h}$ stands for the enthalpy source term of fluid, ${\vec{J}}_{j}$ stands for the diffusion flux of species j, ${\vec{J}}_{i}$ stands for the diffusion flux of species i, $Y_{i}$ stands for the local mass fraction of species i, and $R_{i}$ stands for the reaction net rate of production of specie i.

The effects of gravity, Dufour effect, and surface reaction were disregarded because employing them result in negligible effects [41]. The flow is treated as incompressible because the maximum value of Mach number between all cases is roughly 2 × 10^−3^. Also, the flow is considered as laminar as the maximum value of Reynolds number based on non-reacting flow calculations in all cases is 227, which is less than 500 [42,43].

### 2.3. Numerical Setup and Boundary Conditions

The chemistry is modeled with the aid of a stiff chemistry solver and finite-rate model, accommodating a hydrogen chemistry mechanism of 9 species (namely H_2_, O_2_, N_2_, H_2_O, H_2_O_2_, HO_2_, H, O and OH) and 19 chemical reactions as shown in Table 2 [44]. The SIMPLE algorithm couples the pressure and velocity; The discretization of the transport equations is conducted with the aid of a second-order upwind scheme. The ideal gas mixing law models viscosity and thermal conductivity, while the mixing law computes the specific heat. Furthermore, the mass diffusivity and density were modeled using kinetic theory and incompressible gas law, respectively. The convergence criteria of 10^−6^ is set to all residuals.

The initial inlet temperature of the premixed charge is set at 300 K. The velocity inlet and pressure outlet are selected for the inlet and outlet boundary conditions, respectively. The diffusive flux species and interior surfaces are assumed to be zero and no-slip boundary conditions, respectively. The heat transfer mechanisms considered in this investigation are conduction and radiation. The parameters are computed by means of the area-weighted average technique.

The total heat loss $\left(Q\right)$ from the external surface of micro combustor is computed as follows [45,46]:

(6) $$Q=Q_{con}+Q_{rad\;}\to h\;A_{c}\left(T_{w}-T_{\infty }\right)+\varepsilon \sigma A_{c}\left(T_{w}^{4}-T_{\infty }^{4}\right)$$

$Q_{con}$ and $Q_{rad}$ donate the heat losses by convection and radiation, respectively, h is the natural convection heat transfer coefficient (20 W/m^2^·K), and ε is the emissivity of the solid surface (0.85) [47,48]. $A_{c}$ is the surface area of the outer wall, $T_{w}$ is the temperature of external wall of, $T_{\infty }$ the ambient temperature (300 K), and σ the Stephan–Boltzmann constant (5.67 × 10^−8^ W/ m^2^·K^4^).

The pressure loss $\left(P_{loss\;}\right)$, which is caused by the friction and heating effects, is computed as [49]:

(7) $$P_{loss\;}=P_{in\;}-P_{out\;}$$

where $P_{in\;}$ and $P_{out\;}$ signify the pressure at the inlet and outlet, respectively.

The calculation of the Peclet number (Pe) reads as follows:

(8) $$Pe=\frac{\rho Vd_{h}C_{p}}{\lambda }$$

where ρ is the density, V is the velocity, $d_{h}$ is the hydraulic diameter, and $C_{p}$ is the specific heat, and λ is the thermal conductivity.

The calculations of diffusion $\left(S_{gen}^{diffussion}\right)$, conduction $\left(S_{gen}^{conduction}\right)$, chemical $\left(S_{gen}^{chemical}\right)$ and total $\left(S_{gen}^{total}\right)$ entropy generations [17,50] read as:

(9) $$S_{gen}^{diffussion}=\sum_{i}^{n}\left(\rho RD_{i}\right)\cdot \frac{\nabla \omega_{i}\nabla \chi_{i}}{\chi_{i}}$$

(10) $$S_{gen}^{chemical}=-\sum_{i}^{n}\frac{1}{T}\mu_{i}\gamma_{i}$$

where

(11) $$\mu_{i}={\bar{h}}_{i}^{o}\left(T\right)-T{\bar{s}}_{i}^{o}\left(T\right)+RTln\left(\frac{\chi_{i}P}{P_{atm}}\right)$$

(12) $$S_{gen}^{conduction}=\frac{1}{T^{2}}k_{mix}{\left(\nabla T\right)}^{2}$$

(13) $$S_{gen}^{total}=\int_{V}S_{gen}^{diffussion}dV+\int_{V}S_{gen}^{chemical}dV+\int_{V}S_{gen}^{conduction}dV$$

where R is the gas constant, $D_{i}$ is the mass diffusivity of species *i*, $\omega_{i}$ is the mass fraction of species *i*, $\chi_{i}$ is the mole fraction of species *i*, $\mu_{i}$ is the chemical potential of species *i*, $\gamma_{i}$ is the production rate of species *i*, ${\bar{h}}_{i}^{o}$ is the reference enthalpy of species *i*, ${\bar{s}}_{i}^{o}$ is the reference entropy of species *i*, $P_{atm}$ is the atmospheric pressure, and $k_{mix}$ is the mixture thermal conductivity.

The exergy efficiency is computed using the second law of thermodynamics [51,52,53]. The evaluations of inlet exergy $\left(E_{x}^{in}\right)$ and total exergy losses $\left(E_{x}^{eg}\right)$ read as follows:

(14) $$E_{x}^{in}={\dot{m}}_{fuel\;}\times Q_{LHV\;}$$

and

(15) $$E_{x}^{eg}=E_{x}^{loss}+{\dot{m}}_{inlet\;}\times T_{\infty }\times c_{p,outlet}\times \ln\frac{T_{\infty }}{T_{eg}}$$

where ${\dot{m}}_{fuel\;}$ is the mass flow rate of fuel, $Q_{LHV\;}$ signifies the lower heating value of hydrogen (119.962 MJ/kg) [4], $E_{x}^{loss}$ is the exergy loss, ${\dot{m}}_{inlet\;}$ is the mass flow rate of inlet flow, $c_{p,outlet}$ the specific heat capacity at the combustor outlet, and $T_{eg}$ exhaust gas temperature.

The exergy balance is implemented to calculate the uncounted exergy destruction $\left(E_{x}^{des}\right)$ as follows:

(16) $$E_{x}^{des}=E_{x}^{in}-E_{x}^{eg}$$

The exergy efficiency $\left(\eta_{exergy}\right)$ is evaluated as

(17) $$\eta_{exergy}=\left(1-\frac{E_{x}^{des}}{E_{x}^{in}}\right)\times 100\%$$

The radiation efficiency $\left(\eta_{radiation}\right)$ is calculated as follows:

(18) $$\eta_{radiation}=\left(\frac{Q_{rad}}{{\dot{m}}_{fuel\;}\times Q_{LHV\;}}\right)\times 100\%$$

The wall temperature is computed based on an area-weighted-mean technique $\left(T_{mw}\right)$ as [23]:

(19) $$T_{mw}=\frac{\sum_{i=1}^{n}{T_{i}A}_{i}}{\sum_{i=1}^{n}A_{i}}$$

where $T_{i}$ is the outer wall temperature at cell i, and $A_{i}$ is the outer wall area at cell i.

The wall temperature uniformity $\left(R_{T}\right)$ is evaluated as:

(20) $$R_{T}=\left(\frac{\sum_{i=1}^{n}\left[\left|T_{i}-T_{mw}\right|A_{i}\right]}{T_{mw}\sum_{i=1}^{n}A_{i}}\right)\times 100\%$$

### 2.4. Grid Independence and Model Validation

Conducting a mesh sensitivity analysis is pivotal in numerical investigations to ensure the accuracy of the results with reasonable computational costs. Here, the computational domain of the micro combustor is discretized with three different mesh resolutions of 1,310,165 (Mesh-I), 844,545 (Mesh-II), and 580,698 (Mesh-III) cell numbers. The findings presented here are produced with a 600 mL/min volume flow rate $\left(Q_{v}\right)$ of hydrogen and a unity equivalence ratio.

**Figure 2:**
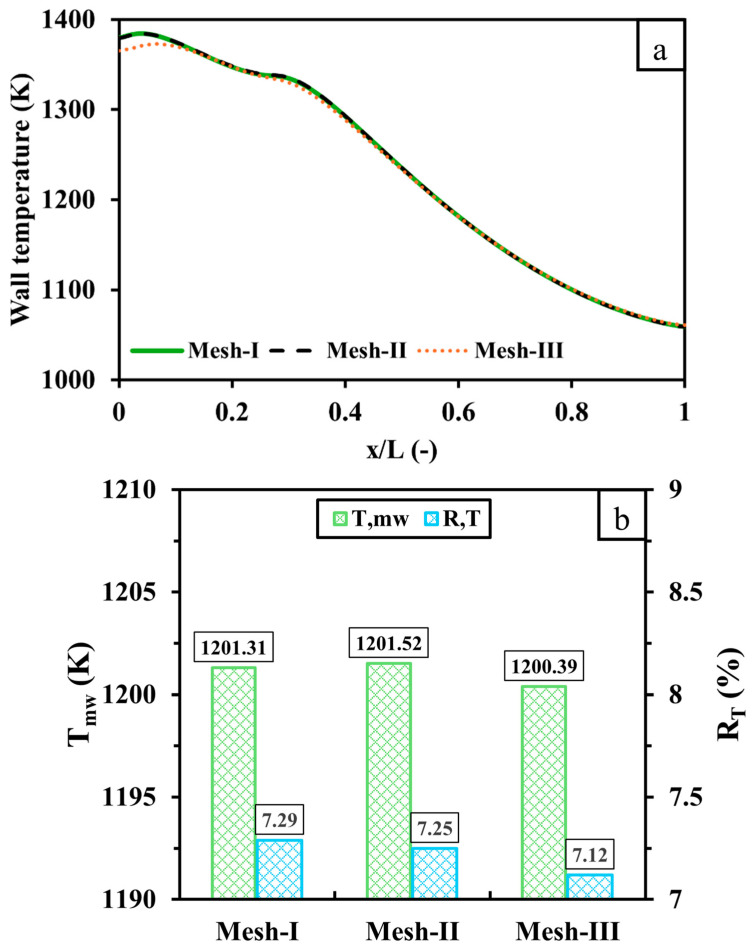
A comparison of (**a**) wall temperature distribution and (**b**) $T_{mw}$ and $R_{T}$ between 1,310,165 (Mesh-I), 844,545 (Mesh-II) and 580,698 (Mesh-III) cell numbers. *x* represents the distance from the inlet, and *L* represents the distance from the inlet and outlet.

Figure 2 compares the three mesh resolutions outlined above with respect to the temperature distribution over the outer wall with respect to the dimensionless length $\left(x/L\right)$, $T_{mw}$, and $R_{T}$. As shown in Figure 2a, the wall temperature variation of Mesh-I is identical to that of Mesh-II. However, the lower-resolution mesh (Mesh-III) under-predicts the wall temperature in the inlet region as opposed to the higher-resolution cases. Figure 2b illustrates that $T_{mw}$ and $R_{T}$ are highly consistent across all cases as the discrepancies of $T_{mw}$ and $R_{T}$ between Mesh-III (Mesh-II) and Mesh-I are 0.92 K (0.21) and 0.17% (0.04%), respectively. Based on this mesh independence analysis, Mesh-II (cell number: 844,545) provides an optimal balance of accuracy and computational efficiency and is thus selected for all subsequent simulations.

**Figure 3:**
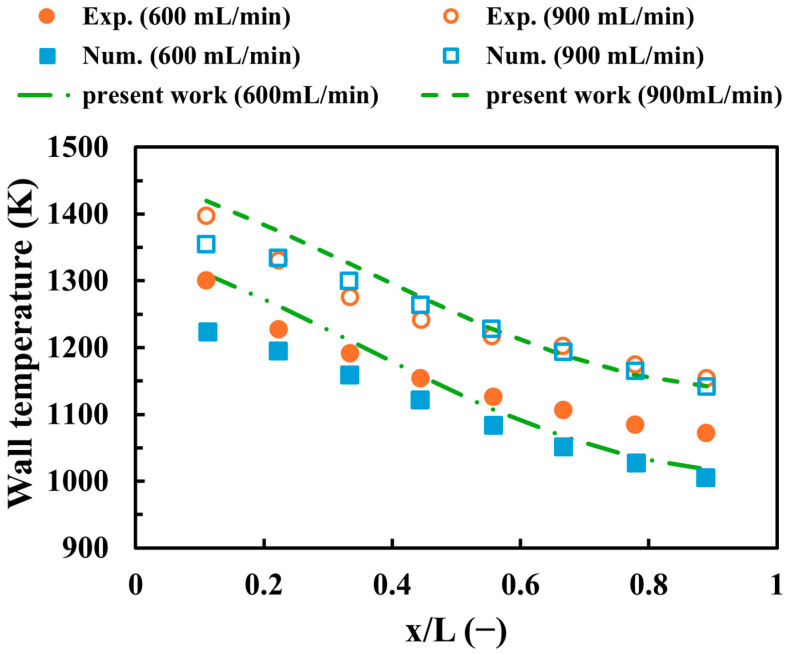
A comparison between the current work and experimental [54] and numerical [55] findings with respect to the centerline wall temperature.

The validation process is an important step for all CFD works to demonstrate the soundness and precision of numerical settings. Thus, Figure 3 displays the validation of the present work against experimental [54] and numerical [55] results with respect to the centerline wall temperature at hydrogen discharge of 600 and 900 mL/min. To ensure an accurate validation process, the length, width, and height of the micro combustor used in the validation are 18 mm, 9 mm, and 3 mm, respectively, including a wall thickness of 0.5 mm, which is the same as the outlined experimental and numerical works. As observed in Figure 3, the variations of the predicted wall temperature of the present work are consistent with both experimental and simulated results, as they show constantly decreasing trends when approaching the outlet. The maximum percentage of errors between the current study and experimental (numerical) data occurs at a 600 mL/min discharge of hydrogen, with a discrepancy of 5.1% (7.03%) in the outlet (inlet) region. The causes of the outlined errors could be attributed to operational or measurement uncertainties for experiments and different mesh resolutions for simulations. In general, these levels of discrepancies are acceptable and, therefore, indicate that the numerical settings and modeling approaches are feasible and viable.

## 3. Results

### 3.1. Effects of the Diamond-Shaped Bifurcated Inner-Tube Structure

The potential of the new micro combustor configuration featuring a diamond-shaped bifurcated inner-tube structure against the conventional combustor is demonstrated here. In addition, this sub-section discusses the effects of the inner structure lengths and the flame path widths for optimization purposes. The test cases investigated in the following discussion are C1, C2, C3, C4, and C5. The C1 case represents the conventional micro combustor, the C2, C3, and C4 cases represent the novel micro combustor design with different diamond-shaped bifurcated configuration lengths, and the C5 case represents the longest inner structure length with wider flame paths. The dimensions corresponding to these cases are listed in Table 1. The hydrogen flow rate and inlet equivalence ratio for all cases are set at 600 mL/min and 1, respectively.

**Figure 4:**
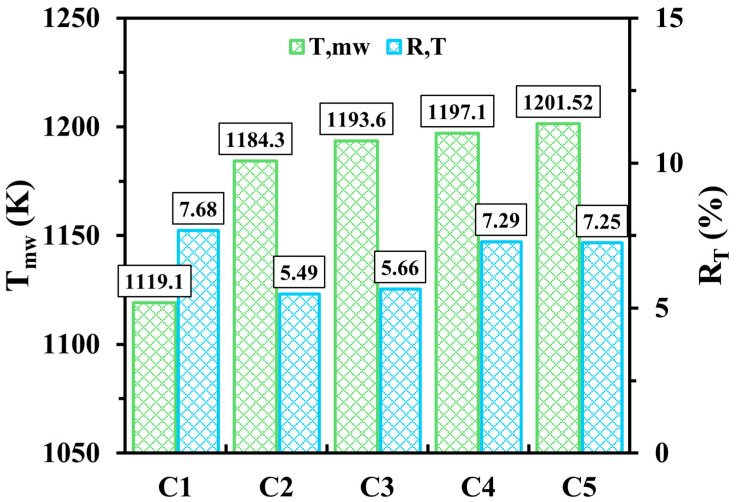
Variations of $T_{mw}$ and $R_{T}$ for C1, C2, C3, C4 and C5 configurations at a hydrogen discharge of 600 mL/min and a unity equivalence ratio.

Figure 4 illustrates the impacts of the novel micro combustor design as opposed to the conventional design, varying internal structure lengths, and flame channel widths on $T_{mw}$ and $R_{T}$. The cases with the newly proposed design are greatly conducive to increasing $T_{mw}$ compared to the conventional design with temperature differences of 65.2, 74.5, 78, and 82.42 K for configurations C2, C3, C4, and C5, respectively, compared to C1. This indicates that the novel configuration, even with various design parameters, exhibits an effective means of promoting the transfer mechanisms of heat from the thermal energy toward the solid walls. The cause of the $T_{mw}$ improvement lies in employing the perforated wall, which acts as a flame holder and consequently elongates the residence time of flow, allowing the outer wall to absorb more heat. Furthermore, $R_{T}$ is higher in C1 as opposed to C2, C3, C4 and C5, revealing that the novel micro combustor configuration in all cases displays more evenly distributed temperature over the outer walls. Figure 4 also shows that as the length of the diamond-shaped bifurcated inner-tube structure transitions from short (C2) to long (C4), both $T_{mw}$ and $R_{T}$ gradually increase, pronouncing an improvement in the absorption rate of thermal energy by walls with the expense of wall temperature uniformity. For the width of the internal heat channel, the trends of $T_{mw}$ and $R_{T}$ indicate that the wider channel (C5) compared to the narrower channel (C4) results in more heat transferring to walls and a slightly more uniform wall temperature due to the high-temperature flow being allowed to travel closer to the walls.

**Figure 5:**
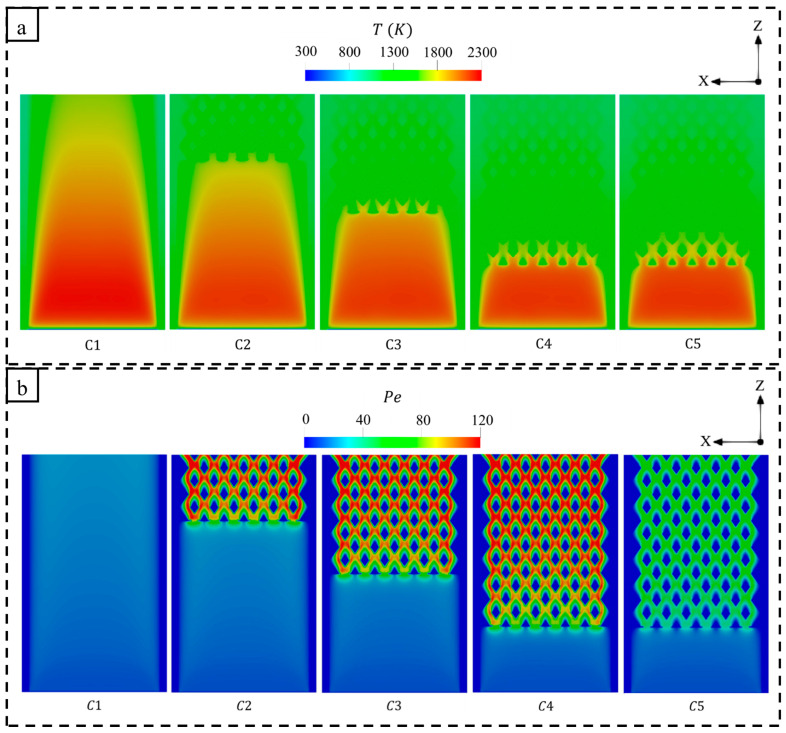
A comparison of (**a**) temperature and (**b**) Peclet (Pe) number variations between different micro combustor configurations.

Figure 5 displays the temperature and Peclet (Pe) number, which is defined as the ratio of advection to diffusion effects [56,57], with respect to the different micro combustor design configurations. As seen in Figure 5a, the high-temperature flame of the conventional micro combustor (C1) narrows when approaching the outlet. In addition, the residence time of the flow of C1 is short due to the absence of the perforated wall. However, employing such a wall holds a high-temperature flame, while the diamond-shaped bifurcated inner-tube structure elongates the dwell time of heat in the micro combustor. For this, the capacity to transfer more thermal energy to the outer walls is improved, resulting in a significant increase in $T_{mw}$ in C2, C3, C4, and C5 in comparison to C1, as illustrated in Figure 4. The length of the novel inner structure design also plays a major role in enhancing the process of heat transfer, as Figure 5a demonstrates that the shortest length (C2), compared to the medium (C3) and long (C4) lengths, allows a large amount of high-temperature flame to propagate for a larger volumetric space, leading to lower thermal energy retention. This significantly improves the uniformity of wall temperature $\left(R_{T}\right)$, whereas the mean wall temperature $\left(T_{mw}\right)$ is relatively lower as the length transitions from short to long (see Figure 4). The cross-sectional plane selected to present the temperature distribution in Figure 5 demonstrates that the temperature similarly varies throughout the domain for the wider (C5) and narrower (C4) flame channels.

Figure 5b shows that the newly proposed designs (C2, C3, C4, and C5) significantly enhance the Pe number, signifying that advection effects dominate over diffusion. It can be observed that a notable increase in the Pe number takes place in the diamond-shaped bifurcated configuration, demonstrating the potential of the proposed design in enhancing the heat transfer process. Thus, elongating the novel configuration facilitates a wider distribution of high Pe across the micro combustor wall. Interestingly, the wider internal flame paths (C5) reduce the effects of advection in comparison to the narrower one (C4) due to decreased hot flow velocities resulting from the larger cross-sectional area of the inner flame channels. This indicates a less effective heat transfer mechanism in C5; however, as shown in Figure 4, the C5 configuration achieves a higher $T_{mw}$ because the flow travels nearer the outer walls.

**Figure 6:**
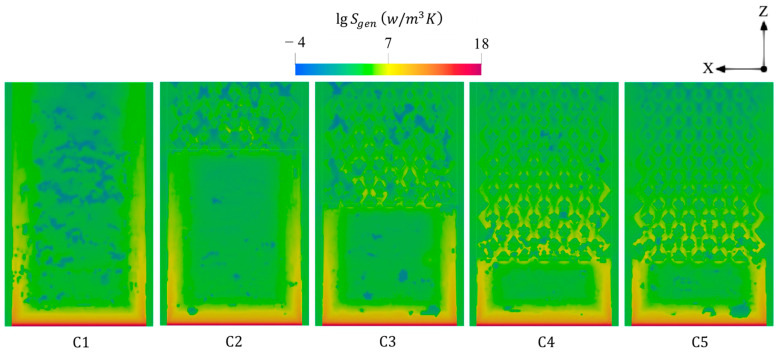
Spatial distributions of total entropy generations under different combustor configurations.

Figure 6 displays the variations of total entropy generations under different combustor configurations at a 600 mL/min volume flow rate of hydrogen and a unity equivalence ratio. The peak of entropy production tends not to be affected by the different design parameters as the peak is localized in the inlet region, possibly due to the onset of combustion, indicating a domination of the chemical entropy generation. However, the conventional design (C1) experiences high entropy generations near the outer walls, whereas employing the diamond-like structure increases the flow resistance in the center of geometry, resulting in high flow irreversibilities; therefore, the proposed design shifts the entropy to be highly produced throughout the inner-tube channels. For this, due to the high number of barriers, elongating the bifurcated inner-tube configuration increases the number of flow barriers and hence enlarges the area occupied by the high entropy generation within this configuration. Nevertheless, widening the inner flame channels (C5) decreases the total entropy generation downstream compared to the narrower one (C4).

**Figure 7:**
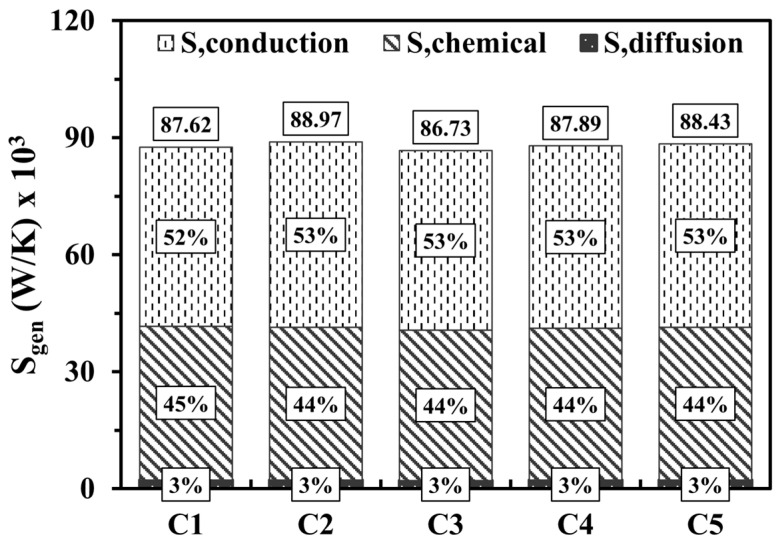
Volume integrations of different entropy generations at various design parameters.

Figure 7 shows the variations of entropy production by conduction, chemical, and diffusion effects at various micro combustor structures. In all cases, the effects of entropy generation by conduction slightly dominate over the chemical effects due to the privilege of the temperature gradients over the combustion intensity. However, even though hydrogen is known as a highly diffusive fuel, the diffusion displays an extremely low contribution to the total entropy generation given the low quantity of hydrogen introduced into the system. Interestingly, altering the design parameters demonstrates a negligible impact on the magnitude of the total entropy generation, which indicates that the different configurations of small-scale applications like micro combustors tend not to increase the irreversibilities and, accordingly, the entropy.

**Figure 8:**
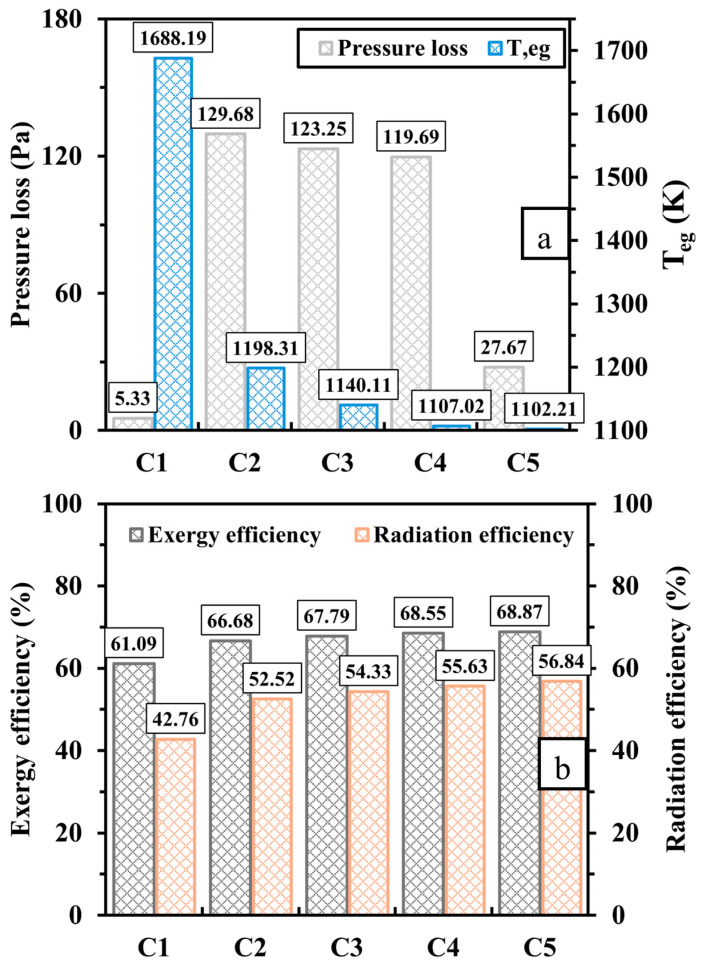
Variations of (**a**) pressure loss and exhaust gas temperature ($T_{eg}$) and (**b**) exergy and radiation efficiencies for different design configurations.

The discrepancies of pressure loss, exhaust gas temperature ($T_{eg}$), and exergy and radiation efficiencies are illustrated in Figure 8. As seen in Figure 8a, the novel design (C2, C3, C4, and C5) experiences a considerable increase in pressure loss as opposed to the conventional one (C1) for a micro-scale application because the perforated wall forms a barrier to the flame, forcing it to flow through smaller volumetric spaces, namely the inner flame channels. This implies that establishing a diamond-shaped structure requires more pumping power. Elongating the length of the proposed configuration progressively reduces the pressure drop, while widening such a structure leads to a notable further reduction. As discussed earlier, the newly proposed design results in an improvement of $T_{mw}$, indicating that more thermal energy is absorbed by walls. Thus, Figure 8a demonstrates that the exhaust gas temperature, which represents the waste thermal energy, is significantly low for the cases with the proposed design, as the differences of $T_{eg}$ between C2, C3, C4, C5, and C1 are 489.88, 548.08, 581.17, and 585.98 K, respectively. Hence, the micro combustor embedded in the diamond-shaped bifurcated inner-tube structure reveals improvements in both exergy and radiation efficiencies, as illustrated in Figure 8b.

### 3.2. Effects of Inlet Velocity

The inlet velocity is paramount to determining the overall system efficiency. Therefore, investigating such an important parameter to demonstrate how it affects the key thermal performance enables the optimization of the initial boundary conditions. Hence, this sub-section discusses the effects of the inlet velocity by means of varying the volume flow rate $\left(Q_{in}\right)$ from 600 to 900 mL/min at a unity equivalence ratio. The configurations selected for all subsequent simulations are C1 and C5, as C5 optimizes the output power density and system efficiencies compared to the newly proposed designs.

**Figure 9:**
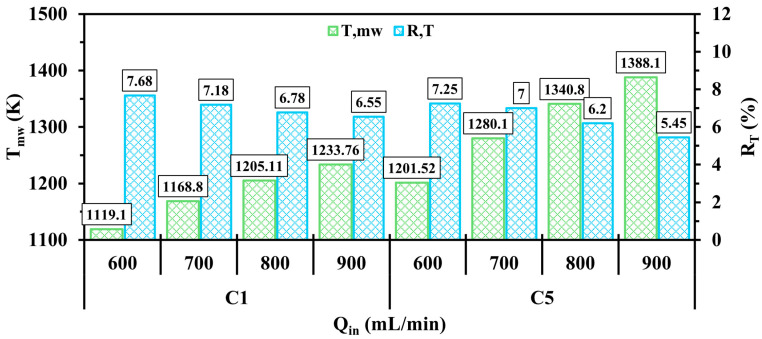
A comparison of $T_{mw}$ and $R_{T}$ in the C1 and C5 configurations under different $Q_{in}$.

The effects of the variation of inlet velocity on $T_{mw}$ and $R_{T}$ are illustrated in Figure 9. As can be seen, increasing the inlet velocity significantly enhances $T_{mw}$ in both configurations, indicating that the outer wall greatly absorbs more thermal energy. This increasing trend is attributed to the system receiving more fuel at higher inlet velocities. Furthermore, $R_{T}$ constantly reduces when $Q_{in}$ approaches 900 mL/min for all test cases, implying a notable improvement in the uniformity of the temperature over micro combustor walls. This improvement suggests that the injection of more hydrogen into the system results in optimal expansion of the hydrogen flame. Since C5 improves $T_{mw}$ and $R_{T}$ in the previous sub-section compared to C1, the level of improvements in the highlighted parameters becomes more pronounced with increasing $Q_{in}$.

**Figure 10:**
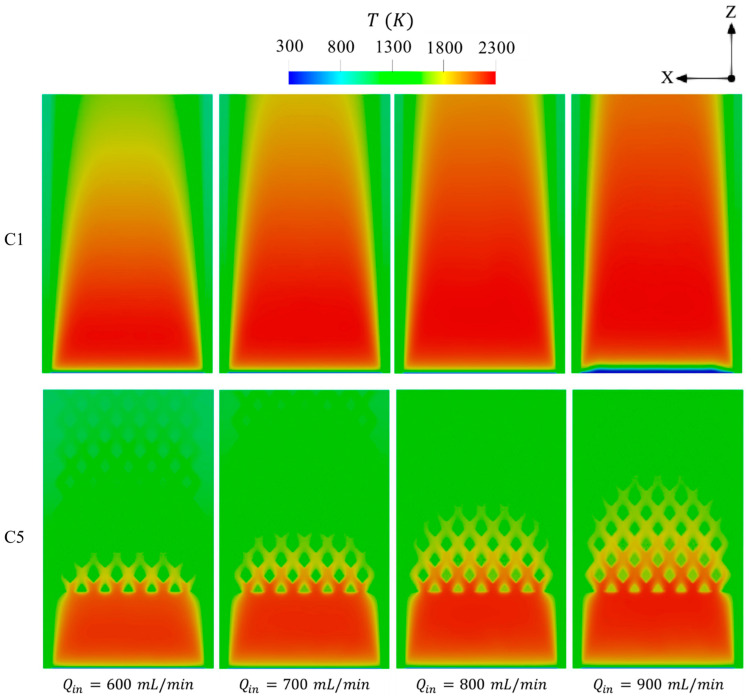
Spatial variations of temperature in the C1 and C5 configurations under different levels of $Q_{in}$.

The effects of inlet velocity on the distributions of temperature are displayed in Figure 10. As observed in Figure 10, elevating $Q_{in}$ leads the area covered by the high temperature to widely distribute across the domain, signifying that introducing more high-flame-speed fuel like hydrogen into the micro combustor enhances the flame to freely propagate over a longer volume. This confirms the improvement in the uniformity of the wall temperature at high velocity, as can be observed in Figure 9. In contrast to C1, the bifurcated diamond-shaped structure in C5 retains a greater thermal energy at higher $Q_{in}$, leading to the transfer of more heat to the walls and, hence, enhancing both $T_{mw}$ and $R_{T}$ as previously observed. In addition to this, holding the hot gases by such a structure also tends to serve as a preheating agent for the inlet flow as the onset of combustion in C5 at $Q_{in}$ of 900 mL/min occurs in the same region as in the other cases with lower $Q_{in}$, whereas combustion in C1 takes place relatively farther downstream under the same condition.

**Figure 11:**
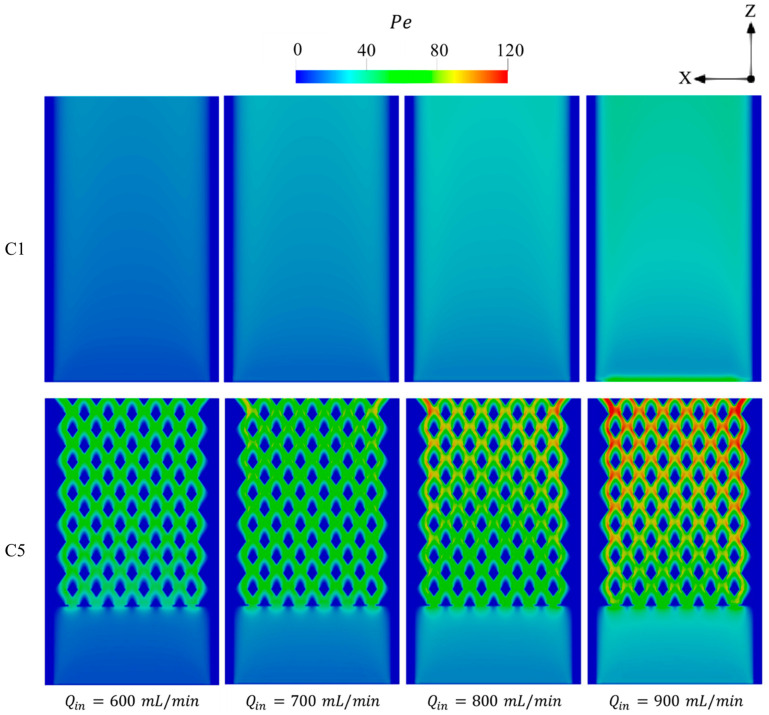
Variations of Pe number in the C1 and C5 configurations under different levels of $Q_{in}$.

Figure 11 shows the variations of Pe number over the micro combustor domain at various $Q_{in}$. In regions occupied by high-temperature flame, the higher $Q_{in}$ conditions result in a gradual promotion of Pe toward the micro combustor’s outlet. This improvement in Pe number is significantly higher in C5, demonstrating the potential of the newly proposed configuration to enhance the advection effects and, accordingly, the heat transfer process. Due to the farther initiation of combustion from the inlet region in C1 configuration at $Q_{in}$ of 900 mL/min, the peak of Pe occurs in the inlet region, which is caused by the high density of the unburnt gases.

**Figure 12:**
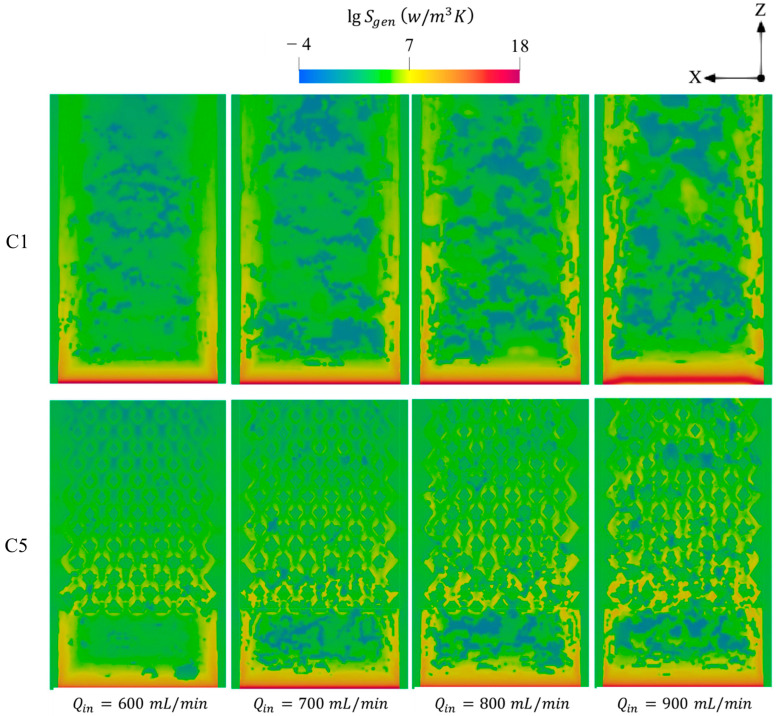
Spatial variations of total entropy productions in the C1 and C5 configurations under different $Q_{in}$.

Figure 12 illustrates the total entropy generated by means of conduction, chemical, and diffusion mechanisms with respect to various $Q_{in}$. For all test cases, the increase in $Q_{in}$ enhances the entropy generations and expands its distribution toward the outlet region. This reveals that injecting a larger quantity of hydrogen into the systems greatly intensifies the combustion rate and also elongates the flame propagation throughout the micro combustor, as shown in Figure 10, implying that the area occupied by the high chemistry rate is enlarged and consequently generates more entropy.

**Figure 13:**
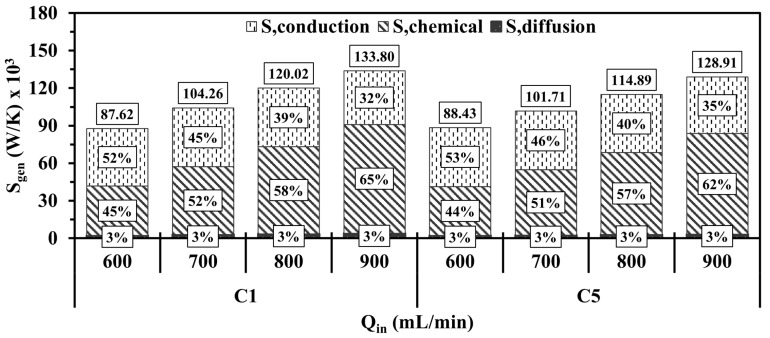
Volume integrations of different entropy generations for C1 and C5 at different $Q_{in}$.

Figure 13 shows the total entropy generation and the contributions of each mechanism in the entropy magnitude under different $Q_{in}$. As observed in Figure 13, elevating $Q_{in}$ in both configurations is conducive to significantly promoting the entropy production by chemistry as $Q_{in}$ of 600, 700, 800, and 900 mL/min in C1 (C5) advances the participation of the chemical effects in the entropy magnitudes by 45%, 52%, 58%, and 65% (44%, 51%, 57% and 62%), respectively. The causes of the gradual increase lie in that a greater amount of energy is introduced into the system at greater $Q_{in}$, thus resulting in a more vigorous chemistry and, hence, more intense combustion [11,29]. Furthermore, the increasingly prominent contribution of the chemistry mechanism progressively lowers the contribution of conduction, whereas the diffusion mechanism accounts for 3% of the total entropy generation with increasing $Q_{in}$, which could be attributed to the utilization of more fuel with high diffusion coefficients like hydrogen. The highlighted observations lead the total entropy generation to increase at higher $Q_{in}$.

**Figure 14:**
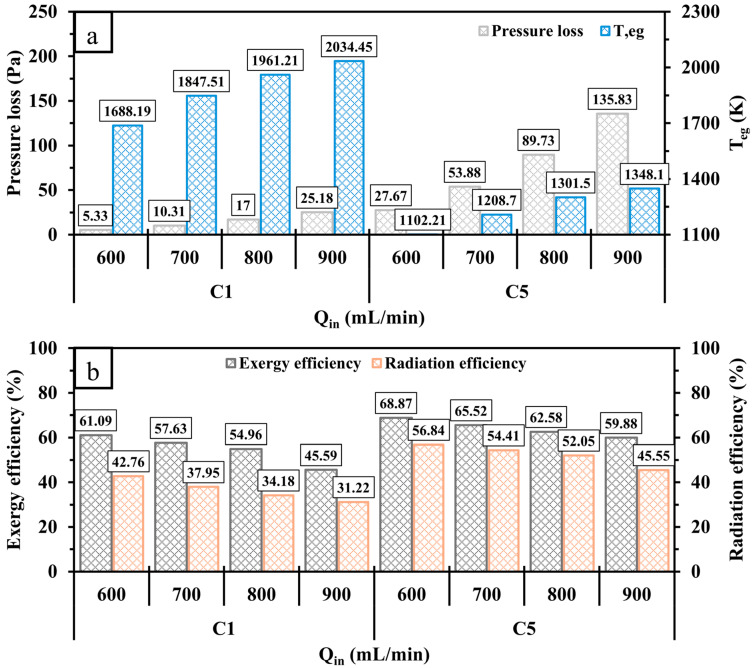
A comparison of (**a**) pressure loss, $T_{eg}$, and (**b**) exergy and radiation efficiencies for C1 and C5 at various $Q_{in}$.

Figure 14 presents the variations in pressure loss, $T_{eg}$, exergy, and radiation efficiencies under different $Q_{in}$ conditions. As depicted in Figure 14a, an increase in $Q_{in}$ in all test cases leads to a gradual rise in pressure loss, indicating the need for more input power in such cases. As $Q_{in}$ approaches 900 mL/min, the relative increase in pressure loss in C5 is higher than in C1, which is caused by the higher rate of collision between the high-velocity flame and the perforated wall. In addition, cases with high velocities exhibit a greater waste of thermal energy as $T_{eg}$ increases with an increase in $Q_{in}$, particularly in C1, which indicates significantly less heat transfer to the outer walls in comparison with C5. For both configurations, the higher waster of thermal energy progressively reduces the exergy and radiation efficiencies, as shown in Figure 14b. It is worth noting that the conventional design (C1) exhibits considerable reductions in the efficiencies compared to C5 due to the greater $T_{eg}$. Feeding the system with a higher amount of hydrogen at a higher $Q_{in}$ considerably improves both $T_{mw}$ and $R_{T}$; however, the variations in the outlined efficiencies imply that the micro combustor appears to have constraints in its capacity to accommodate a substantial amount of energy.

### 3.3. Effects of Inlet Equivalence Ratio

The equivalence ratio in combustion-based applications $\left(\Phi \right)$ is one of the most important factors, as it is considered a critical parameter in controlling the degree of complete combustion. Thus, this sub-section presents the effects of Φ ranging from 0.6 to 1.2 on thermal parameters at $Q_{in}$ of 600 mL/min.

**Figure 15:**
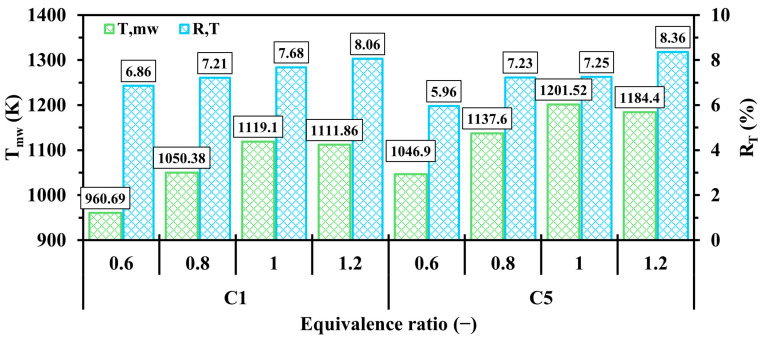
Variations of $T_{mw}$ and $R_{T}$ in C1 and C5 configurations with respect to different Φ.

Figure 15 compares $T_{mw}$ and $R_{T}$ at various Φ ranging from 0.6 to 1.2. For both configurations, the $T_{mw}$ pattern fluctuates as it significantly increases from the fuel-lean to stoichiometric conditions (Φ = 0.6 to 1) and then decreases when approaching the fuel-rich condition (Φ = 1.2). This highlights that the cases of $\left(\Phi <1\right)$ exhibit an effective means of reducing $T_{mw}$ due to the low quantity of hydrogen supplied to the system leading to less thermal energy release during combustion, whereas the cases of $\left(\Phi >1\right)$ suffers from the low availability of oxygen, resulting in a lower consumption rate of fuel compared to the stoichiometric condition. This pronounces that the unity Φ optimizes the mean wall temperature parameter. As shown in Figure 15, $R_{T}$ consistently increases from low to high Φ, indicating that higher Φ values lead to a more uneven temperature distribution along the micro combustion walls.

**Figure 16:**
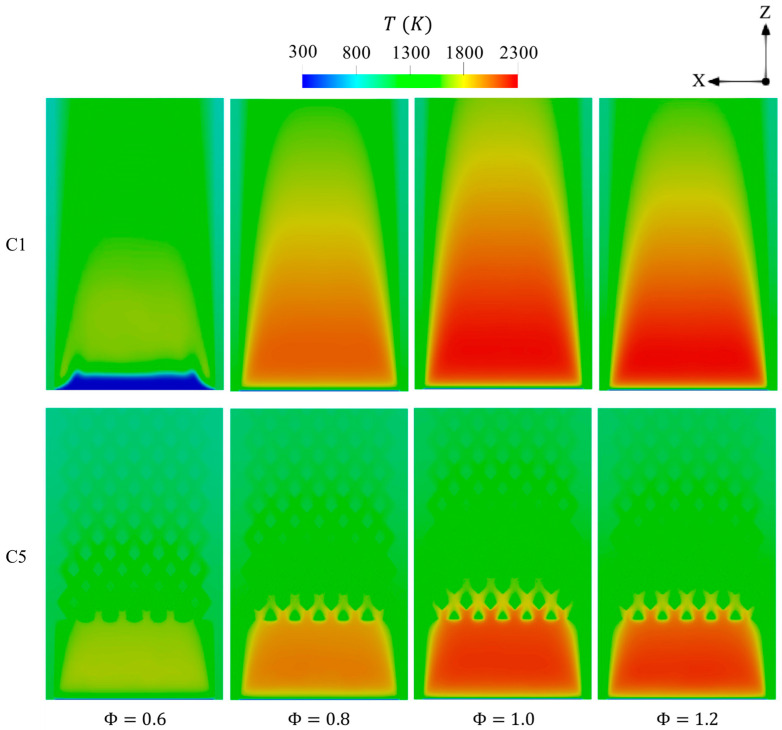
Spatial variations of temperature throughout the geometry in the C1 and C5 configurations with respect to different Φ.

Figure 16 illustrates the effects of varying Φ from 0.6 to 1.2 on the distribution of temperature over the cross-sectional plane of the micro combustor. Φ = 1 optimally balances hydrogen and oxidizer in all test cases, resulting in the maximum temperature, as shown in Figure 16. However, the $\left(\Phi <1\right)$ cases display a remarkably low temperature due to the ignition of a low amount of energy during combustion, whereas the $\left(\Phi >1\right)$ case reduces the maximum temperature as opposed to that of stoichiometric condition because it suffers from a low chemistry rate as the system is fed with insufficient oxygen. In contrast to C5, the C1 configuration at Φ of 0.6 exhibits a farther initiation of combustion from the inlet, which could indicate combustion failure at leaner conditions. However, the preheating factor resulting from heat retention by the proposed inner structure in C5 leads the combustion to occur in the same area as richer conditions $\left(\Phi >0.6\right)$. This likely suggests that the flammability limit becomes wider with the new design.

**Figure 17:**
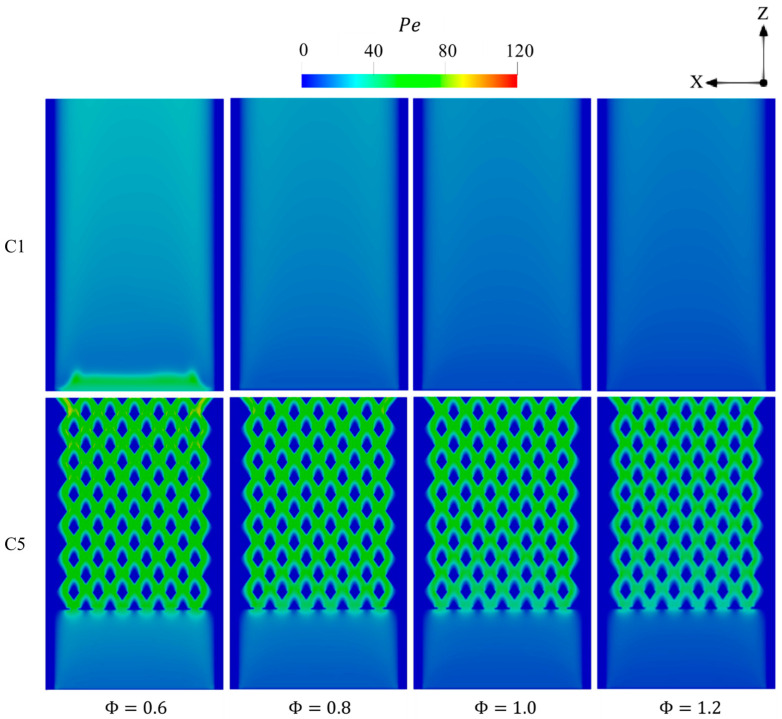
Spatial variations of Pe throughout the geometry in the C1 and C5 configurations with respect to different Φ.

Figure 17 demonstrates that elevating Φ in both configurations results in a reduction in Pe in the combustion regions. As hydrogen is known for its high diffusivity, higher Φ values increase the hydrogen concentration and, therefore, dominate the diffusion effects over that of advection, which consequently reduces Pe. As noticed in Figure 16, the combustion is shifted downstream in C1 at Φ=0.6 as the peak of Pe is found to be in the inlet area due to the high density of unburnt incoming mixture.

**Figure 18:**
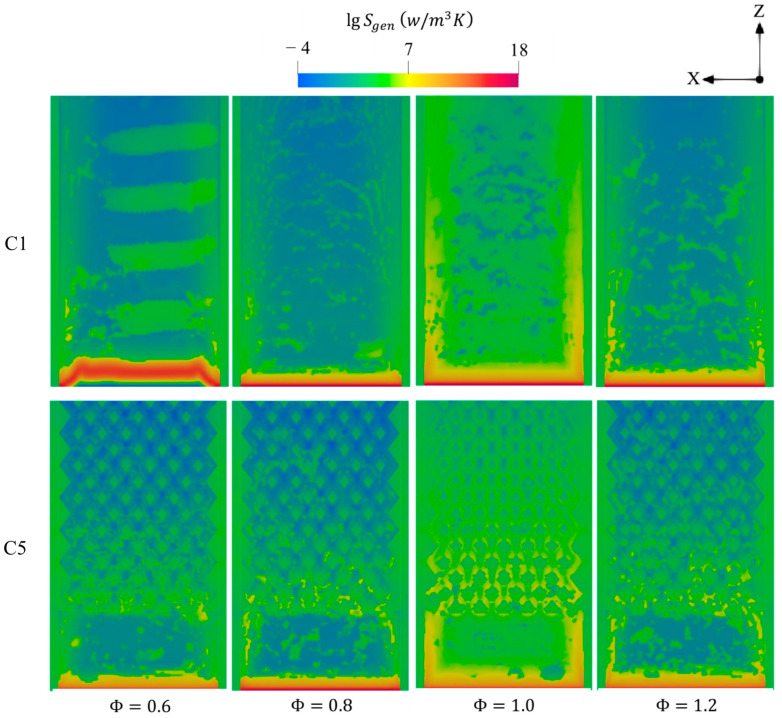
Contours of total entropy productions for C1 and C5 under different Φ.

Figure 18 depicts the effects of Φ on the entropy generations. In all cases, the variation of entropy over the micro combustor significantly expands as Φ transitions from lean to stoichiometric conditions and then notably shrinks when approaching the fuel-rich condition. This reveals the greater intense combustion rate in Φ=1 enhances all the mechanisms utilized to account for the entropy generations and, therefore, elevates the total system irreversibilities. The $\left(\Phi >1\right)$ case exhibits a wider variation of entropy compared to the cases of $\left(\Phi <1\right)$, thereby indicating the greater heat release rate in the former case. This observation confirms the findings shown in Figure 16, as the temperature of combustion is remarkably higher in Φ=1.2 as opposed to the cases of Φ=0.8 and 0.6.

**Figure 19:**
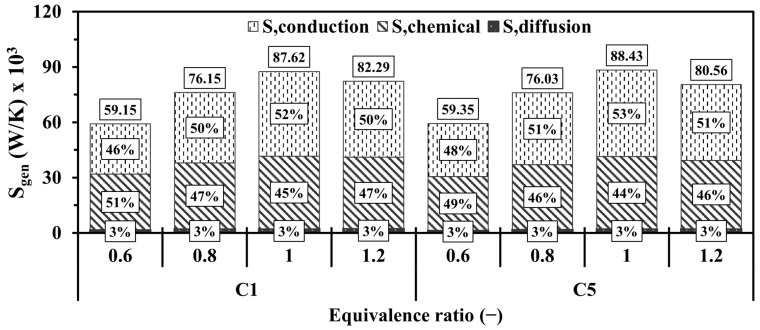
Volume integrations of different entropy generations in the configurations of C1 and C5 at different Φ.

Figure 19 provides further insight into the magnitude of the entropy and the contribution of the conduction, chemical, and diffusion effects with respect to various Φ. In general, the higher magnitude of entropy production in both configurations occurs at the equivalence ratios of 1, 1.2, 0.8, and 0.6, respectively. Surprisingly, the vigorous chemistry rate in the case of the unity Φ promotes the participation of the conduction mechanism at a higher degree compared to that of chemistry. This suggests that the highlighted case notably intensifies combustion and, therefore, releases more thermal energy, thereby enlarging the temperature gradients between the flow and walls and leading to dominating the effects of the conduction in increasing the entropy production. The new design (C5) demonstrates slightly higher contributions of entropy generations in comparison to the conventional one (C1), indicating greater temperature gradients.

**Figure 20:**
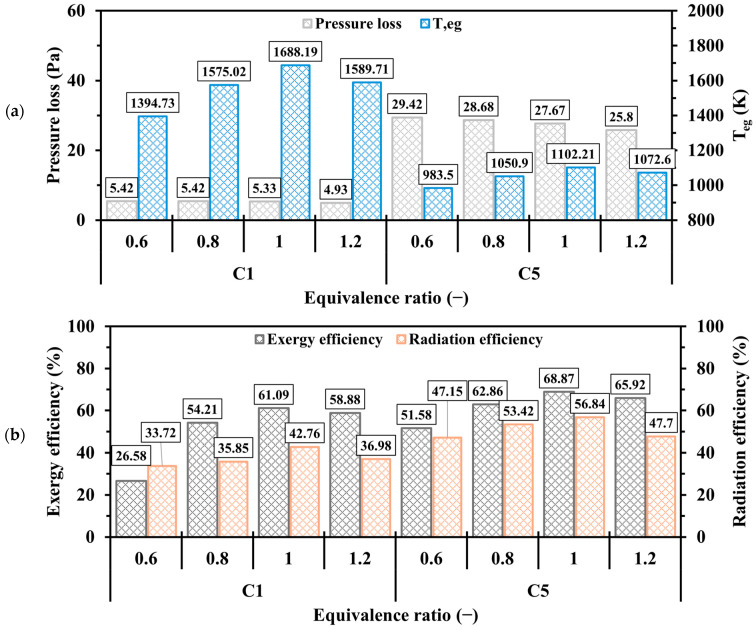
Discrepancies of (**a**) pressure loss, $T_{eg}$, (**b**) exergy and radiation efficiencies in the C1 and C5 configurations at various Φ.

Figure 20 depicts the effects of Φ on the pressure loss, $T_{eg}$, exergy, and radiation efficiencies. The variation in Φ has a negligible effect on pressure drop, with minimal differences observed between lean and rich conditions. Figure 20a shows that $T_{eg}$ in all cases increases as Φ transitions from 0.6 to 1, followed by a decrease as Φ approaches 1.2. This trend arises because excess oxygen (in the case of Φ<1) surpasses the needed quantity, whereas excess hydrogen (for Φ>1) requires more oxygen to chemically react with. It is true that the unity Φ results in the greatest waste of thermal energy compared to other cases; however, it achieves the highest exergy and radiation efficiencies, as shown in Figure 20b, indicating that the stoichiometric condition optimizes the performance parameters.

## 4. Conclusions

This study investigates a newly proposed hydrogen-fueled micro combustor configuration featuring a diamond-shaped bifurcated diamond-shaped structure. The 3D numerical analysis assesses the impact of the proposed design on the thermal performance. A parametric study to optimize system efficiency is conducted by varying the inner-tube length, flame channel width, volume flow rate, and inlet equivalence ratio. The main findings are as follows:

- The proposed configuration notably enhances mean wall temperature and its uniformity across the external surface compared to the conventional design. Furthermore, the variation in Pe numbers across the geometry implies that the new design remarkably enhances advection effects and, consequently, the heat transfer process. For this, the mean exhaust gas temperature is lowered, while both exergy and radiation efficiencies show notable improvements.
- Extending the length of the diamond-shaped inner-tube configuration enhances the mean temperature over the solid walls, although it reduces the uniformity of temperature across the walls. Moreover, the region with high Pe variations is concentrated in the diamond-shaped section; hence, its elongation shifts the thermal dynamics toward advection dominance over diffusion. This reduces pressure loss and mean exhaust gas temperature and, however, advances exergy and radiation efficiencies. Widening the flame channels in this extended structure further improves thermal performance.
- Escalating the hydrogen volume flow rate results in a greater mean wall temperature and more temperature uniformity across the walls. In addition, it amplifies the Pe number, pressure loss, and mean exhaust gas temperature because a higher energy input is supplied to the system. Nevertheless, the micro combustor’s limited capacity to effectively utilize large energy volumes causes exergy and radiation efficiencies to decrease as the flow rate increases. The entropy generation is highly increased when the inlet velocity is increased, owing essentially to the considerable enhancement of the chemical entropy mechanism.
- In contrast to the stoichiometric condition, variations in the equivalence ratio reveal that both lean and rich fuel mixtures lead to lower system performance due to insufficient hydrogen or oxygen in the premixed charge, respectively. This indicates that an equivalence ratio of unity optimizes system efficiency and output power density. However, the unity equivalence ratio case achieves the peak entropy production due to the large difference in temperature gradients (conduction entropy mechanism) between the heat and walls.

In conclusion, the newly proposed design configuration enhances the heat transfer mechanisms from combustion to walls. However, it is important to conduct further investigations to address some practical issues as follows:

- The high auto-ignition temperature of hydrogen could be one of the practical limitations of using hydrogen in such applications. Thus, examining the effects of using catalysts to lower the ignition temperature is critical.
- The high wall temperature could damage the wall material. Thus, an investigation into employing thermal carrier coatings on the wall material is needed to ensure the sustainability of the micro combustor.

## Figures and Tables

**Table 1:** Dimensions of the new design configuration. L, θ and C stand for length, angle and case, respectively.

Variables		Value (mm)				
		C 1	C 2	C 3	C 4	C 5
Length (mm)	L1	18	18	18	18	18
	L2	11	11	11	11	11
	L3	4	4	4	4	4
	L4	NA	5	9	13	13
	L5	NA	1.25	1.25	1.25	1.25
	L6	NA	2	2	2	2
	L7	NA	0.9	0.9	0.9	1.8
	L8	NA	0.9	0.9	0.9	0.9
Angle (degree)	θ	NA	106.26	106.26	106.26	106.26

**Table 2:** Reversible chemical reactions of H_2_-air combustion, where $A_{k}$, $\beta_{k}$, and $E_{k}$ stand for pre-exponential factor of reaction rate, activation energy of the reaction, and temperature exponent, respectively. Note that M represents a third body efficiency.

Reactions	$A_{k}$ (m kmol s)	$\beta_{k}$	$E_{k}$ (J/mol)
O_2_ + H = OH + O	5.10 × 10^13^	−0.82	6.91 × 10^7^
2. H_2_ + O = OH + H	1.80 × 10^7^	1.00	3.70 × 10^7^
3. H_2_ + OH = H_2_O + H	1.20 × 10^6^	1.30	1.52 × 10^7^
4. OH + OH = H_2_O + O	6.00 × 10^6^	1.30	0.00
5. H_2_ + O_2_ = OH + OH	1.70 × 10^10^	0.00	2.0 × 10^8^
6. H + OH + M = H_2_O + M ^a^	7.50 × 10^17^	−2.60	0.00
7. O_2_ + M = O + O + M	1.90 × 10^8^	0.50	4.001 × 10^8^
8. H_2_ + M = H + H + M ^b^	2.20 × 10^9^	0.50	3.877 × 10^8^
9. H + O_2_ + M = HO_2_ + M ^c^	2.10 × 10^12^	−1.00	0.00
10. H + O_2_ + O_2_ = HO_2_ + O_2_	6.70 × 10^13^	−1.42	0.00
11. H + O_2_ + N_2_ = HO_2_ + N_2_	6.70 × 10^13^	−1.42	0.00
12. HO_2_ + H = H_2_ + O_2_	2.50 × 10^10^	0.00	2.90 × 10^6^
13. HO_2_ + H = OH + OH	2.50 × 10^11^	0.00	7.90 × 10^6^
14. HO_2_ + O = OH + O_2_	4.80 × 10^10^	0.00	4.20 × 10^6^
15. HO_2_ + OH = H_2_O + O_2_	5.00 × 10^10^	0.00	4.20 × 10^6^
16. HO_2_ + HO_2_ = H_2_O_2_ + O_2_	2.00 × 10^9^	0.00	0.00
17. H_2_O_2_ + M = OH + OH + M	1.30 × 10^14^	0.00	1.905 × 10^8^
18. H_2_O_2_ + H = H_2_ + HO_2_	1.70 × 10^9^	0.00	1.57 × 10^7^
19. H_2_O_2_ + OH = H_2_O + HO_2_	1.0 × 10^10^	0.00	7.50 × 10^6^

^a^ Enhancement factors: H_2_O = 20.0. ^b^ Enhancement factors: H_2_O = 6.0, H = 2.0, and H_2_ = 3.0. ^c^ Enhancement factors: H_2_O = 21.0, H_2_ = 3.3, O_2_ = 0.0, and N_2_ = 0.0.

## Data Availability

The data presented in the study are included in the article; further inquiries can be directed to the corresponding author.

## Abbreviations

Pe	Peclet number, $\left(-\right)$
$\vec{v}$	Vector velocity, $\left(m\cdot s^{-1}\right)$
$Q_{con}$	Heat losses by convection, $\left(W\right)$
$Q_{rad}$	Heat losses by radiation, $\left(W\right)$
E	Total total energy of the fluid, $\left(J\cdot {kg}^{-1}\right)$
h	Natural convection heat transfer coefficient, $\left(W\cdot m^{-2}\cdot K^{-1}\right)$
$R_{i}$	Reaction net rate of production of species i
$k_{eff}$	Effective thermal conductivity, $\left(W\cdot m^{-2}\cdot K^{-1}\right)$
$h_{j}$	Specific nthalpy of species *j*, $\left(J\cdot {kg}^{-1}\right)$
$S_{h}$	Source term of enthalpy, $\left(W\cdot m^{-3}\right)$
$A_{i}$	Outer wall area of cell *i*, $\left(m^{2}\right)$
$A_{c}$	Surface area of the outer wall, $\left(m^{2}\right)$
${\vec{J}}_{j}$	Diffusion flux of species j, $\left(mol\cdot m^{-2}\cdot s^{-1}\right)$
$Y_{i}$	Local mass fraction of species *i*, $\left(-\right)$
$S_{gen}^{total}$	Total entropy generation, $\left(W\cdot K^{-1}\right)$
$S_{gen}^{chemical}$	Entropy generation as a result of chemical reaction, $\left(W\cdot K^{-1}\right)$
$S_{gen}^{conduction}$	Entropy generation as a result of heat conduction, $\left(W\cdot K^{-1}\right)$
$S_{gen}^{diffussion}$	Entropy generation as a result of mass diffusion, $\left(W\cdot K^{-1}\right)$
$k_{mix}$	Mixture thermal conductivity, $\left(W\cdot m^{-1}K^{-1}\right)$
${\bar{h}}_{n}^{o}$	Reference enthalpy of species *i*, $\left(J\cdot {kg}^{-1}\right)$
${\bar{s}}_{n}^{o}$	Reference entropy of species *i*, $\left(J\cdot {kg}^{-1}\cdot K^{-1}\right)$
$E_{x}^{in}$	Inlet exergy, $\left(W\right)$
$E_{x}^{eg}$	Total exergy losses, $\left(W\right)$
$E_{x}^{des}$	Uncounted exergy destruction, $\left(W\right)$
$E_{x}^{loss}$	Energy loss from the combustion exhaust gas, $\left(W\right)$
P	Pressure, $\left(Pa\right)$
$P_{atm}$	Atmospheric pressure, $\left(101,325\;Pa\right)$
$T_{eg}$	Exhaust gas temperature, $\left(K\right)$
$T_{w}$	Temperature of external wall, $\left(K\right)$
$T_{w,m}$	Area-weighted-mean wall temperature, $\left(K\right)$
$T_{i}$	Outer wall temperature of cell *i*, $\left(K\right)$
$T_{\infty }$	Ambient temperature, $\left(K\right)$
$R_{T}$	Wall temperature uniformity, $\left(\%\right)$
$D_{i}$	Mass diffusivity of species *i*, $\left(m^{2}\cdot s^{-1}\right)$
${\dot{m}}_{inlet\;}$	Mass flow rate of inlet flow, $\left(kg\cdot s^{-1}\right)$
${\dot{m}}_{fuel\;}$	Mass flow rate of fuel, $\left(kg\cdot s^{-1}\right)$
V	Velocity, $\left(m\cdot s^{-1}\right)$
$C_{p\;}$	Specific heat capacity, $\left(J\cdot {kg}^{-1}K^{-1}\right)$
R	Gas constant, $\left(J\cdot {kg}^{-1}K^{-1}\right)$
$Q_{LHV\;}$	Lower heating value, $\left(MJ\cdot {kg}^{-1}\right)$
Greek letters	
ρ	Mixture gas density, $\left(kg\cdot m^{-3}\right)$
$\chi_{i}$	Mole fraction of species, *i*
$\vec{\tau }$	Viscous stress, $\left(N\cdot m^{-2}\right)$
${\vec{\tau }}^{\prime }$	Reynolds stress, $\left(N\cdot m^{-2}\right)$
$\mu_{i}$	Chemical potential of species *i*, $\left(J\cdot {kg}^{-1}\right)$
$\omega_{i}$	Mass fraction of species, *i*
σ	Stephan–Boltzmann constant, $\left(5.67\times 10^{-8}\;W\cdot m^{-2}K^{-4}\right)$
Φ	Equivalence ratio, $\left(-\right)$
λ	Thermal conductivity, $\left(W\cdot m^{-1}K^{-1}\right)$
ε	Emissivity of the solid surface.
$\gamma_{i}$	Production rate of species *i*, $\left(mol\cdot L^{-1}\cdot s^{-1}\right)$
$\eta_{exergy}$	Exergy efficiency, $\left(\%\right)$
$\eta_{radiation}$	Radiation efficiency, $\left(\%\right)$
